# HPRS: hierarchical potential-based reward shaping from task specifications

**DOI:** 10.3389/frobt.2024.1444188

**Published:** 2025-02-10

**Authors:** Luigi Berducci, Edgar A. Aguilar, Dejan Ničković, Radu Grosu

**Affiliations:** ^1^ Cyber-Physical Systems Group, Computer Engineering, TU Wien, Vienna, Austria; ^2^ Center for Digital Safety and Security, AIT Austrian Institute of Technology GmbH, Vienna, Austria

**Keywords:** robotics, robot learning, reinforcement learning, reward shaping, formal specifications

## Abstract

The automatic synthesis of policies for robotics systems through reinforcement learning relies upon, and is intimately guided by, a reward signal. Consequently, this signal should faithfully reflect the designer’s intentions, which are often expressed as a collection of high-level requirements. Several works have been developing automated reward definitions from formal requirements, but they show limitations in producing a signal which is both effective in training and able to fulfill multiple heterogeneous requirements. In this paper, we define a task as a partially ordered set of safety, target, and comfort requirements and introduce an automated methodology to enforce a natural order among requirements into the reward signal. We perform this by automatically translating the requirements into a sum of safety, target, and comfort rewards, where the target reward is a function of the safety reward and the comfort reward is a function of the safety and target rewards. Using a potential-based formulation, we enhance sparse to dense rewards and formally prove this to maintain policy optimality. We call our novel approach hierarchical, potential-based reward shaping (HPRS). Our experiments on eight robotics benchmarks demonstrate that HPRS is able to generate policies satisfying complex hierarchical requirements. Moreover, compared with the state of the art, HPRS achieves faster convergence and superior performance with respect to the rank-preserving policy-assessment metric. By automatically balancing competing requirements, HPRS produces task-satisfying policies with improved comfort and without manual parameter tuning. Through ablation studies, we analyze the impact of individual requirement classes on emergent behavior. Our experiments show that HPRS benefits from comfort requirements when aligned with the target and safety and ignores them when in conflict with the safety or target requirements. Finally, we validate the practical usability of HPRS in real-world robotics applications, including two sim-to-real experiments using F1TENTH vehicles. These experiments show that a hierarchical design of task specifications facilitates the sim-to-real transfer without any domain adaptation.

## 1 Introduction

Reinforcement learning (RL) is an increasingly popular method for training autonomous agents to solve complex tasks in sophisticated environments (Mnih et al., 2015; Lillicrap et al., 2016; Silver et al., 2017). At the core of RL lies the reward function, a user-provided signal that guides the learning process by rewarding or penalizing the agent’s actions. As autonomous agents become increasingly capable and are expected to operate in real-world environments, they are asked to solve tasks with a growing number of requirements, each with different levels of importance and sometimes opposing objectives. Since the reward function must capture all the desired aspects of the agent’s behavior, significant research effort has been invested in reward shaping over the past years (Ng et al., 1999; Laud and DeJong, 2003).

There are two major challenges in defining meaningful rewards, which are best illustrated with an autonomous-driving (AD) application. The first arises from mapping numerous requirements into a single scalar reward signal. In AD, there are more than 200 rules that need to be considered when assessing the course of action (Censi et al., 2019). The second challenge stems from the highly non-trivial task of determining the relative importance of these different requirements. In this realm, there are a plethora of regulations ranging from safety and traffic rules to performance, comfort, legal, and ethical considerations.

In order to address these challenges and train the policy to tackle tasks composed by many heterogeneous requirements, we introduce a novel framework which automatically defines the reward function from the user-defined formal requirements in a systematic fashion. Although former approaches based on formal languages define a task as the combination of safety and liveness formulas (Jothimurugan et al., 2019; Icarte et al., 2018), we introduce a specification language to describe a task as a composition of *safety*, *target*, and *comfort* requirements. This formulation captures a broad class of problems and induces a natural ordering among requirements, according to the class of membership: safety has the highest priority, followed by target that guides goal achievement and, finally, comfort, which are secondary and optional requirements.

In contrast to existing reward design approaches that rely on manual tuning or learning of reward models (Christiano et al., 2017), we propose a fully automated approach based on formal task specifications. In particular, we leverage the partial order of requirements and the quantitative evaluation of each of them to derive a reward function that inherently captures the interdependence between different classes of requirements. Unlike multi-objective approaches that produce Pareto-optimal solutions, the proposed requirement class prioritization induces an unambiguous trajectory ranking. The HPRS shaping optimizes all the requirements simultaneously by combining them in one multivariate multiplicative objective. Moreover, we characterize the proposed reward function as a potential-based signal, which allows us to provide theoretical guarantees on HPRS soundness (Ng et al., 1999). Finally, in contrast to logic-based approaches, which adopt robustness to compute the reward on complete or partial transition sequences (Li et al., 2017; 2018; Balakrishnan and Deshmukh, 2019), we provide a dense reward signal. In this manner, HPRS avoids delaying reward computation over time and mitigates the temporal credit-assignment problem, where a deferred reward is not efficiently propagated to the preceding transitions.

Our approach builds on top of the following four major components:

• An expressive formal specification language capturing classes of requirements that often occur in control tasks.
• An additional specification layer, allowing to group sets of requirements and define priorities among them.
• An automatic procedure for generating a reward, following the order relation among the different requirements.
• A training pipeline with integrated domain adaptation to mitigate sim-to-real transfer.

The advantage of our approach is the seamless passage from task specifications to learning optimal control policies that satisfy the associated requirements while relieving the engineer from the burden of manually shaping rewards.

In the experimental evaluation, we investigate the following research questions (RQs):

• **RQ1**: How does HPRS compare with existing logic-based and multi-objective shaping approaches in producing an effective training signal for reinforcement learning?
• **RQ2**: How do policies trained with HPRS effectively capture the hierarchical structure of task requirements?
• **RQ3**: What is the influence of HPRS’s hierarchical structure on the emergent behavior, particularly concerning the less prioritized comfort requirements?
• **RQ4**: How does HPRS demonstrate practical usefulness in real-world robotics applications?

We answer the RQs by evaluating HPRS on four continuous-control benchmarks (cart-pole, lunar lander, bipedal walker classic, and hardcore) and four physics-simulated environments consisting of two autonomous driving scenarios (stand-alone and follow the leader) and two robot locomotion tasks (ant and humanoid). Our experiments show competitive results compared to state-of-the-art approaches, outperforming logic-based approaches in terms of training efficiency and alignment with the intended behavior and multi-objective approaches in terms of generality and robustness to different parameterizations. Moreover, we deploy the trained policies on F1TENTH racecars (O’Kelly et al., 2020), demonstrating the practical usability of the proposed approach in non-trivial real-world robotics systems.

### 1.1 Contributions

In this paper, we introduce HPRS for RL from a set of formal requirements, proposing a learning pipeline to produce control policies amenable to real-world robotics applications. An initial draft of HPRS appeared in Berducci et al. (2021) and was used as background material in Berducci and Grosu (2022).

Here, we extend the HPRS theory, implementation, experimental evaluation, and applicability as follows:

1. We reframe the HPRS theory and associated proofs to general unconstrained MDP, including theorems and proofs of the main results.
2. We implement HPRS in auto-shaping, the first library for reward shaping from hierarchical formal task specifications, which is integrated with state-of-the-art frameworks (Raffin et al., 2019) and monitoring tools (Ničković and Yamaguchi, 2020).
3. We evaluate HPRS on a broader set of simulated tasks with a large number of comfort requirements, presenting an extended evaluation and ablation studies of the proposed approach.
4. We present a training pipeline with domain adaptation to deal with real-world uncertainties and added two real-world experiments using F1TENTH racecars to showcase the practical applicability.

**FIGURE 1:**
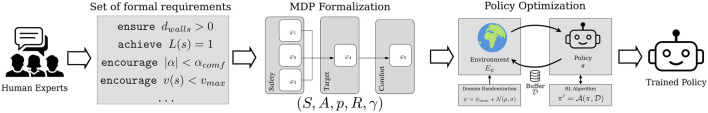
Proposed optimization pipeline: the process begins with human experts formalizing the task requirements as a set of formal specifications. These requirements are processed through an automatic procedure to define an MDP with a dense and informative reward signal that reflects the prioritized hierarchy of requirements. This shaped MDP can then be used with any reinforcement learning algorithm to train an agent capable of performing the prescribed task while adhering to the specified priorities.

The proposed framework in Figure 1 requires minimal user intervention in the specification phase and automates the problem definition and policy optimization. To support the robust transfer of the learned policy to real-world applications, we integrate a domain randomization module (Tobin et al., 2017), which generates a diverse but plausible set of training environments. The approach is proven to be effective in various benchmarks and capable of handling the transfer to real-world applications.

### 1.2 Motivating example

We motivate our work with an *autonomous-driving task*: a *car* drives around a track delimited by *walls* by controlling its speed and steering angle. The car is considered to have completed a *lap* when it drives around the track and reaches its starting position.

The task has seven *requirements*: (1) the car shall never collide against the walls; (2) the car shall complete one lap in bounded time; (3) the car shall drive in the center of the track; (4) the car shall maintain a speed above a minimum value; (5) the car shall maintain a speed below a maximum value; (6) the car shall drive with a comfortable steering angle; (7) the car shall send smooth control commands to the actuators.

**FIGURE 2:**
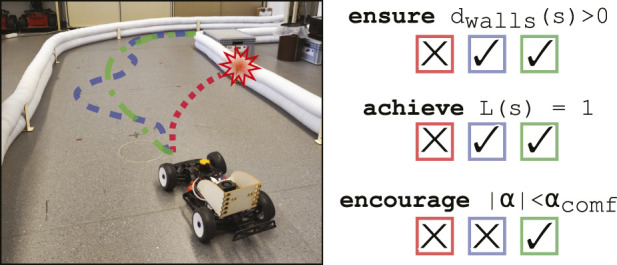
Ranking of behaviors for the driving example: the task is defined by safety, target, and comfort requirements, which together induce a ranking of trajectories: **(red)** the car crashes into the wall, violating the safety requirement. **(blue)** The car safely progresses toward lap completion but exhibits unnecessary jerk, resulting in poor comfort. **(green)** The car satisfies both safety and target requirements while also optimizing comfort. This illustrates the natural ranking based on the requirements’ classes, with safety taking the highest priority, followed by target achievement and, lastly, comfort.

A moment of thought reveals that these requirements might interfere with each other. For example, a car always driving above the minimum speed (requirement 4) while steering below the maximum angle (requirement 6) would have a limited turn curvature. Any track layout containing a turn with a curvature larger than this limit would result in a collision, thus violating requirement 2. Furthermore, if the policy drives with high-frequency saturated actuation only (i.e., bang–bang), the resulting behavior is uncomfortable for the passengers and not transferable to real hardware because of actuator limitations. Figure 2 shows various intended and unintended behaviors.

In this example, it also becomes evident that some requirements must have precedence over others. We consider *safety* as one of those requirements that fundamentally constrain the policy behavior, such as a catastrophic collision against the walls. Therefore, we interpret a safety violation as one compromising the validity of the entire episode. Lap completion (requirement 2) is also a unique requirement that represents the agent’s main objective, or *target*, and, in essence, its whole reason to be. After the safety requirement, this comes next in the hierarchy of importance. Explicitly, it means that we are willing to sacrifice the rest of the requirements (requirements 3–7) in order to complete a collision-free lap around the track. These requirements are, therefore, soft constraints that should be optimized as long as they do not interfere with safety and the target. We call them *comfort*.

In summary, we pose the following research question in this paper: *is there a principled way to shape an effective reward that takes into account all the task requirements in the order of importance mentioned above?* In the rest of this paper, we will illustrate the necessary steps leading to a positive answer, considering the motivating example.

## 2 Related work

RL has emerged as a powerful framework for decision-making across diverse domains, including robotics. The design and specification of reward functions are critical for ensuring that RL agents achieve the desired outcomes efficiently and safely. Consequently, a reward design has been extensively studied across various research communities. This section provides an overview of key research directions addressing this challenge, including reward shaping, RL with temporal logic, multi-objective RL, and formalization of hierarchically structured requirements. Moreover, we highlight how our approach differs from the existing literature in these areas.

### 2.1 Reward shaping

Specifying reward functions for decision-making algorithms is a long-studied problem in the RL community. A poorly designed reward might not capture the actual objective and result in problematic or inefficient behaviors (Amodei et al., 2016). Therefore, shaping the reward helps in effectively steering the RL agent toward favorable behaviors (Ng et al., 1999; Laud and DeJong, 2003). Reward shaping aims in improving the training efficiency of reinforcement learning by modifying the reward function (Eschmann, 2021; Devidze et al., 2021). A particularly notable approach, which will be central in this work, is potential-based reward shaping (PBRS) (Ng et al., 1999). PBRS modifies the original reward using the difference of potential functions to accelerate learning. The characterization of potential as state-dependent functions leads to preserving policy optimality (Ng et al., 1999), and it has been later connected to the initialization of Q-values (Wiewiora, 2003). PBRS continues to be particularly effective in environments with sparse or delayed rewards, improving convergence rates in tasks such as robotic control (Duan et al., 2016), and it represents a fundamental result of reinforcement learning theory (Sutton, 2018), based on which we are going to develop our methodology.

### 2.2 RL with temporal logic

Temporal logic (TL) is a well-suited formalism for specifying complex temporal behaviors in an unambiguous way. For this reason, several works adopt TL to specify reward functions in RL. Although some works focus on multitasking specifications, advancing techniques for task decomposition (Jothimurugan et al., 2021; Toro Icarte et al., 2018; Camacho et al., 2017; Jothimurugan et al., 2019), or study formulations that are tailored for formally specified tasks (Fu and Topcu, 2014; Jones et al., 2015; Icarte et al., 2018; Li et al., 2018), we consider the body of research closer to the problem of reward shaping from formal task specification. The main works develop methods to exploit the quantitative semantics of signal temporal logic (STL) and its variants to systematically derive a reward. STL quantitative semantics (Maler and Nickovic, 2004) are traditionally non-cumulative and non-Markovian since the evaluation depends on the entire trajectory. This makes them difficult to integrate with contemporary RL algorithms, which are developed under these assumptions. For these reasons, approaches that delay the credit assignment to evaluate the complete (Li et al., 2017) or partial sequences of transitions (Balakrishnan and Deshmukh, 2019) have only partially mitigated the underlying problems. The specification of the task as a monolithic formula and the use of quantitative semantics suffer from poor usability in RL due to locality and masking of competing terms (Mehdipour et al., 2019). To overcome these issues, we define a task as the composition of different requirements and develop an automatic methodology to provide a dense reward at every step. This approach is more in line with cumulative RL formulations used in robotics and completely agnostic to the learning algorithm.

### 2.3 Multi-objective RL

Multi-objective RL (MORL) studies reinforce learning algorithms to optimize multiple and often conflicting objectives (Roijers et al., 2013; Hayes et al., 2022). Several algorithms have been proposed to learn single or multiple policies (Liu et al., 2015; Tutsoy, 2021). There exist several techniques to combine multiple rewards into a single scalar value via scalarization, such as linear or non-linear projections (Natarajan and Tadepalli, 2005; Barrett and Narayanan, 2008; Van Moffaert et al., 2013). Other approaches formulate structured rewards by imposing or assuming a preference ranking on the objectives and finding an equilibrium among them (Gábor et al., 1998; Shelton, 2001; Zhao et al., 2010; Abels et al., 2019; Mehdipour et al., 2020). However, the problem of specifying preferences and studying the tradeoff among different classes of requirements remain a challenge. To overcome this issue, we exploit the natural interpretation of the requirement classes to impose an unambiguous interpretation of task satisfaction without the need to deal with Pareto-optimal solutions. From this task semantics, we propose an automatic reward-shaping methodology that enforces it into a scalar reward without requiring tedious tuning of weights. Compared to widely adopted linear scalarizations (Mehdipour et al., 2020; Brys et al., 2014), we adopt a multivariate formulation to combine individual requirements in a multiplicative way (Russell and Norvig, 2020) to capture the inter-class dependence of requirements. Therefore, instead of relying on the arbitrary choice of weights for each requirement, we define a systematic methodology to produce a reward signal. For completeness, in the experimental phase, we compare our approach to various instances of the multi-objective method adopted in Brys et al. (2014) and show the impact of having an arbitrary choice of static weights.

### 2.4 Hierarchically structured requirements

Partial ordering of requirements into a hierarchy has been proposed before in different settings. In this context, *hierarchical* refers to the structure of the task itself, where requirements are prioritized based on their importance, and it differs from the meaning that *hierarchical* has in the literature on hierarchical control framework (Barto and Mahadevan, 2003). The *rulebook* formalism uses a hierarchy of requirements for evaluating behaviors produced by a planner (Censi et al., 2019) without addressing the problem of learning a control policy from it. More recent works propose to synthesize a policy with optimal control and enforce hard constraints through CBF (Xiao et al., 2021) or with receding horizon planning (Veer et al., 2023). However, although they assume perfect knowledge of the environment dynamics and focus on planning for autonomous driving, our work, to the best of our knowledge, is the first to use hierarchical task specifications with model-free reinforcement learning for robotics control. Complementary approaches use hierarchical specifications with inverse RL (Puranic et al., 2021), learning dependencies among formal requirements from demonstrations. However, although they learn dependencies from data, we infer them from requirement classes and use them in reward shaping.

## 3 Methods

In this section, we present our main contribution: *A method for automatically generating a reward-shaping function from a plant definition and a set of safety, target, and comfort requirements*. In order to make this method accessible, we first introduce a *formal language* allowing formulation of the requirements mentioned above. Our method performs the following steps:

• *Step 1:* infers the priority among different requirements based on the class of membership and formulates a task as a partially ordered set of requirements.
• *Step 2:* extends the plant definition to an MDP by adding a sparse-reward signal to reflect task satisfaction and the episode termination conditions.
• *Step 3:* enrich the reward with a continuous HPRS signal by hierarchically evaluating the individual requirements.

The resulting training signal can then be used by any RL algorithm and integrated with domain adaptation to deal with sim-to-real transfer. We discuss and demonstrate its applicability in the experimental section.

### 3.1 Requirement specification language

We formally define a set of expressive operators to capture requirements that often occur in control problems. Considering atomic predicates p≐f(s) ≥ 0 over observable states s ∈ S , we extend existing task-specification languages (e.g., SpectRL (Jothimurugan et al., 2021)) and define the requirements as follows:

φ≐achievep | conquerp | ensurep | encouragep.

Commonly, a task can be defined as a set of requirements from three basic classes: *safety*, *target*, and *comfort*. Safety requirements, of the form ensurep , are associated with an invariant condition p . Target requirements, of the form achievep or conquerp , formalize the one-time or the persistent achievement of a goal within an episode, respectively. Finally, comfort requirements, of the form encourage p , introduce the soft satisfaction of p , as often as possible, without compromising task satisfaction.

Let $\tau =\left(s_{0},a_{1},s_{1},a_{2},\ldots \right)$ denote an episode of |τ|=t steps, and let T be the set of all such traces. Each requirement φ induces a Boolean function σ: T → B, evaluating whether an episode τ ∈ T satisfies the requirement φ . We define the requirement satisfaction function σ as follows:

$$\begin{array}{lll} \sigma \;\left(achieve\;p,\tau \right) & \; & \text{ iff }\;\exists i\leq t\text{ s.t. }f\left(s_{i}\right)\geq 0,\\ \sigma \;\left(conquer\;p,\tau \right) & \; & \text{ iff }\;\exists i\leq t\text{ s.t. }\forall j\geq i,f\left(s_{j}\right)\geq 0,\\ \sigma \;\left(ensure\;p,\tau \right) & \; & \text{ iff }\;\forall i\leq t\text{ s.t. }f\left(s_{i}\right)\geq 0,\\ \sigma \;\left(encourage\;p,\tau \right) & \; & \text{ iff }\;true. \end{array}$$

Example: let us consider the motivating example and formally specify its requirements. The state $s=\left(x,y,\theta ,v,\dot{\theta }\right)$ consists of x,y,θ for the car position and heading in global coordinates, and v and $\dot{\theta }$ are the car speed and rotational velocities, respectively. The control action is a=(ν,α), where ν denotes the desired speed and α denotes the steering angle*.*

We first define (1) $d_{\text{walls}}:S\to R$ , a distance function that returns the distance of the car to the closest wall; (2) L:S→[0,1] , a lap progress function that maps the car position to the fraction of track that has been driven from the starting position; (3) $d_{\text{center}}:S\to R$ , a distance function that returns the distance of the car to the centerline; (4) the maximum deviation from the centerline $d_{\text{comf}}$ that we consider tolerable; (5) the maximum steering angle $\alpha_{\text{comf}}$ that we consider being comfortable to drive straight; (6) the minimum and maximum speed $v_{\min},v_{\max}$ that define the speed limits; (7) the maximum tolerable change in controls Δa that we consider to be comfortable. Then, the task can be formalized with the requirements reported in Table 1 *.*

**TABLE 1:** Formalized requirements for driving example: the task is formalized as a set of formulas. *Req1* ensures that a minimum distance from walls is maintained; *Req2* specifies completing a lap; *Req3* encourages tracking the center-line within tolerance; *Req4–5* encourage maintaining a velocity within limits; *Req6–7* encourage comfortable controls with small steering angles and smooth changes.

Req id	Formula id	Formula
Req1	$\varphi_{1}$	$ensure\;d_{\text{walls}}\left(s\right)>0$
Req2	$\varphi_{2}$	achieveL(s)=1.0
Req3	$\varphi_{3}$	$encourage\;d_{\text{center}}\left(s\right)\leq d_{\text{comf}}$
Req4	$\varphi_{4}$	$encourage\;v\geq v_{\min}$
Req5	$\varphi_{5}$	$encourage\;v\leq v_{\max}$
Req6	$\varphi_{6}$	$encourage\;|\alpha |\leq \alpha_{\text{comf}}$
Req7	$\varphi_{7}$	encourage|a|≤Δa

### 3.2 A task as a partially ordered set of requirements

We formalize a task by a partially ordered set of formal requirements, Φ , assuming that the target is unique and unambiguous. Formally, $\Phi =\Phi_{S}⊎\Phi_{T}⊎\Phi_{C}$ , such that

$$\begin{array}{c} \Phi_{S}≔\left\{\varphi \;|\;\varphi ≐ensure\;p\right\}\\ \Phi_{C}≔\left\{\varphi \;|\;\varphi ≐encourage\;p\right\}\\ \Phi_{T}≔\left\{\varphi \;|\;\varphi ≐achieve\;p\;\vee \;\varphi ≐conquer\;p\right\} \end{array}$$

The target requirement is required to be unique $\left(|\Phi_{T}|=1\right)$ .

We use a very natural interpretation of importance among the class of requirements, which considers decreasing importance from safety, to target, and to comfort requirements.

Formally, this natural interpretation of importance defines a (strict) partial order relation ≺ on Φ , which is defined as follows:

$$\varphi ≺\varphi^{\prime }\text{ iff }\left(\varphi \in \Phi_{S}\wedge \varphi^{\prime }\notin \Phi_{S}\right)\vee \left(\varphi \in \Phi_{T}\wedge \varphi^{\prime }\in \Phi_{C}\right)$$

The resulting pair (Φ,≺) forms a partially ordered set of requirements and defines our task. Extending the satisfaction semantics to a set, we consider a task accomplished when all of its requirements are satisfied, as follows:

$$\sigma \left(\Phi ,\tau \right)\text{ iff }\forall \varphi \in \Phi ,\;\sigma \left(\varphi ,\tau \right)$$ (1)

The priority among the class of requirements induces an ordering on trajectories or *rank* (Veer et al., 2023). The intuition of episode rank based on our requirements is the following: an episode fully satisfying all the classes of requirements has the highest rank (i.e., rank=1 ) and an episode only satisfying safety and target has a lower rank but is still higher than an episode only satisfying safety; finally, an episode violating all the classes of requirements has the lowest rank (i.e., $rank=2^{3}$ ).

Definition 1 Given an episode τ and a task specification (Φ,≺) , where $\Phi =\Phi_{S}⊎\Phi_{T}⊎\Phi_{C}$ , we define the rank of the episode over N=3 classes as follows:

$$rank\left(\Phi ,\tau \right)=2^{N}-\overset{N-i}{\underset{i=1}{\sum }}\sigma \left(\Phi_{C_{i}},\tau \right)$$

### 3.3 MDP formalization of a task

We assume that the plant (environment controlled by an autonomous agent) is given as $E\;=\;\left(S,S_{0},A,P\right)$ , where S is the set of states, $S_{0}$ is the set of initial states, A is the set of actions, and $P\left(s^{\prime }|s,a\right)$ is its dynamics, that is, the probability of reaching state $s^{\prime }$ by performing action a in state s .

Given an episodic task (Φ,≺) over a bounded time horizon T , our goal is to automatically extend the environment E to a Markov decision process (MDP) $M\;=\;\left(S,S_{0},A,P,R,T\right)$ . To this end, we define $R\left(s,a,s^{\prime }\right)$ , the reward associated with the transition from state s to $s^{\prime }$ under action a , to satisfy (Φ,≺) .

#### 3.3.1 Episodes

An episode ends when its task satisfaction is decided either through a safety violation, timeout, or goal achievement. The goal achievement evaluation depends on the target operator adopted: for achievep , the goal is achieved when visiting at time t ≤ T , a state $s_{t}$ that satisfies p ; for conquerp , the goal is achieved if there is a time i ≤ T such that p is satisfied for all $s_{i},\;i\;\leq \;t\;\leq \;T$ .

#### 3.3.2 Base reward

Given the task (Φ,≺) , we first define a sparse reward that incentivizes achieving the goal. Let the property of the unique target requirement be p≐f(s)≥0 . Then,

$$R\left(s,a,s^{\prime }\right)=\left\{\begin{array}{cc} 1\; & \;\text{if }f\left(s^{\prime }\right)\geq 0\\ 0\; & \;otherwise \end{array}\right.$$

The rationale behind this choice is that we aim to teach the policy to reach the target and stay there as often as possible. For achievep , R maximizes the probability of satisfying p . For conquerp , there is an added incentive to reach the target as soon as possible and stay there until T .

The associated MDP is, in principle, solvable with any RL algorithm. However, although the sparse base reward R can help solve simple tasks, where the target is easily achieved, it is completely ineffective in more complex control tasks.

### 3.4 Hierarchical potential-based reward shaping

Here, we introduce the main contribution of this work, our hierarchical potential-based reward shaping (HPRS). Introducing a novel design to produce a dense reward signal based on the hierarchy of requirements, we aim to continuously provide feedback during training, guiding the agent toward the task satisfaction.

We assume the predicates p(s) ≐ f(s) ≥ 0 to be not trivially satisfied in all the states s ; otherwise, they can be omitted by the specification. Each signal is then bounded in [l,u] for l<0<u . We also define the negatively saturated signal $f_{-}\left(s\right)\;=\;min\left(0,f\left(s\right)\right)$ and the two following signals:

$$c\left(p,s\right)≐1-\frac{f_{-}\left(s\right)}{l},\;b\left(p,s\right)≐1_{\geq 0}\left(f\left(s\right)\right),$$

where $1_{\geq 0}\left(\cdot \right)$ is an indicator function of non-negative numbers. Both c and b are bounded in [0,1] , where 1 denotes the satisfaction of p and 0 denotes its largest violation. However, although c is a continuous signal, b is discrete, with values in {0,1} .

Using the signals c and b , we now define the individual score r for each requirement φ ∈ Φ as follows:

$$r\left(\varphi ,s\right)=\left\{\begin{array}{cc} b\left(p,s\right)\; & \text{if}\;\varphi \in \Phi_{S}\\ c\left(p,s\right)\; & \text{otherwise} \end{array}\right..$$ (2)

Definition 2 Let (Φ,≺) be a task specification. Then, the hierarchical potential function is defined as follows:

$$\Psi \left(s\right)=\underset{\varphi \in \Phi }{\sum }\left(\underset{\varphi^{\prime }:\varphi^{\prime }≺\varphi }{\prod }r\left(\varphi^{\prime },s\right)\right)\cdot r\left(\varphi ,s\right).$$ (3)

**FIGURE 3:**
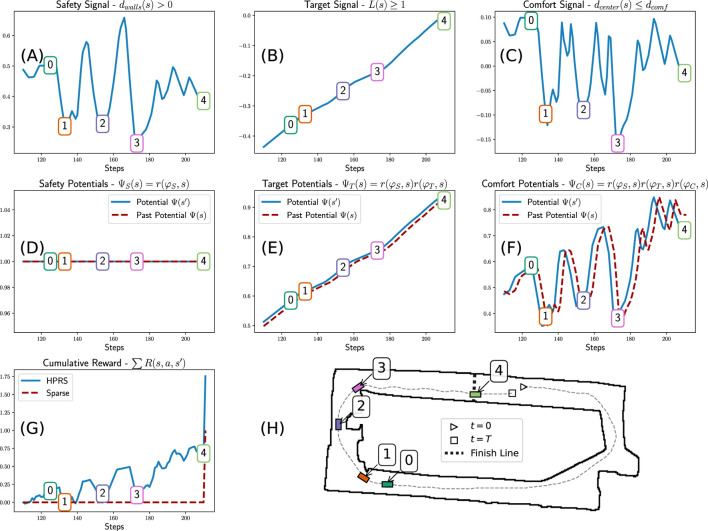
Hierarchical potential-based reward shaping: Simulation of a car driving a full lap (**H**) showing trajectory (*dashed line*) and key timesteps (*numbered boxes*): (0) approaching a turn, (1) entering a turn, (2) passing an obstacle, (3) exiting a turn, and (4) final straight. **(A–C)** Robustness signals for the **(A)** safety, **(B)** target, and **(C)** comfort requirements. **(D–F)** Potentials using the hierarchical weights based on membership class. **(G)** Sum of rewards for sparse and our HPRS reward. The following patterns emerge: safety is consistently maintained at 1. Target is incrementally achieved. Comfort is satisfied as long as it does not conflict with safety and target. The comfort signal decreases near turns and obstacles, and its contribution in the reward increases with target. The overall training signal is much more dense than the sparse base reward.

This potential function is a weighted sum over all requirement scores r(φ,s) . The weight of r(φ,s) is the product of the scores $r\left(\varphi^{\prime },s\right)$ of all the requirements $\varphi^{\prime }$ that are strictly more important than φ . A visual representation of the signals composing the reward for our motivating example is depicted in Figure 3.

Example: let us consider the motivating example and unpack the potential term defined in Equation 3 *.* For each of the seven requirements, we define the score terms, as shown in Equation 2:

$$r\left(\varphi_{1},s\right),r\left(\varphi_{2},s\right),r\left(\varphi_{3},s\right),r\left(\varphi_{4},s\right),r\left(\varphi_{5},s\right),r\left(\varphi_{6},s\right),r\left(\varphi_{7},s\right).$$

Let $\Phi =\Phi_{S}⊎\Phi_{T}⊎\Phi_{C}$ represent the set of requirements defining the task. We expand the inner terms of Equation 3, weighting each score term based on the scores of higher-priority requirements:

$$\Psi_{\varphi_{1}}\left(s\right)=r\left(\varphi_{1},s\right),$$

$$\Psi_{\varphi_{2}}\left(s\right)=r\left(\varphi_{1},s\right)\cdot r\left(\varphi_{2},s\right),$$

$$\Psi_{\varphi_{i}}\left(s\right)=r\left(\varphi_{1},s\right)\cdot r\left(\varphi_{2},s\right)\cdot r\left(\varphi_{i},s\right),\;\forall i=3,\ldots ,7.$$

Each term is weighted according to the task hierarchy semantics. Safety is the highest priority, so the potential for $\varphi_{1}$ is unweighted. Target follows safety, so the potential for $\varphi_{2}$ is weighted by safety. Comfort is the lowest priority, so the potentials for all comfort requirements $\varphi_{i}\;\left(i=3,\ldots ,7\right)$ are weighted by both safety and target. Finally, the overall potential is defined as the sum of the intermediate terms:

$$\Psi \left(s\right)=\Psi_{\varphi_{1}}\left(s\right)+\Psi_{\varphi_{2}}\left(s\right)+\Psi_{\varphi_{3}}\left(s\right)+\Psi_{\varphi_{4}}\left(s\right)+\Psi_{\varphi_{5}}\left(s\right)+\Psi_{\varphi_{6}}\left(s\right)+\Psi_{\varphi_{7}}\left(s\right).$$

The potential is thus a *multivariate signal that combines the scores with multiplicative terms* (Russell and Norvig, 2020), according to the ordering defined in the task (Φ,≺) . A linear combination of scores, as typical in multi-objective scalarization, would assume independence among objectives and would not be expressive enough to capture their interdependence (Russell and Norvig, 2020). Crucially, the weights dynamically adapt at every step as well, according to the satisfaction degree of the requirements.

Corollary 1 The optimal policy for the MDP $M^{\prime }$ , where its reward $R^{\prime }$ is defined with HPRS as

$$R^{\prime }\left(s,a,s^{\prime }\right)=R\left(s,a,s^{\prime }\right)+\Psi \left(s^{\prime }\right)-\Psi \left(s\right)$$ (4)

is also an optimal policy for the MDP M with reward R .

This corollary shows that HPRS preserves the policy optimality for the considered undiscounted episodic setting. It follows by the fact that Ψ:S → R is a potential function that is proved to preserve the policy optimality (Ng et al., 1999). For completeness, we report the proof of this standard result in the following.

Proof: consider the MDP $M\;=\;\left(S,S_{0},A,P,R,T\right)$ , and let $M^{\prime }$ be the MDP obtained by transforming the reward with the hierarchical potential shaping described in Equation 4.

Let $\pi_{M}^{⋆}$ denote the optimal policy for M , which maximizes the optimal action-value function $Q_{M}^{⋆}$ as follows:

$$\pi_{M}^{⋆}\left(s\right)\in \underset{a\in A}{\text{arg max}}Q_{M}^{⋆}\left(s,a\right).$$

The optimal action-value function $Q_{M}^{⋆}$ satisfies the Bellman equation, which, for the undiscounted episodic MDP considered in this work (i.e., discount γ=1 ), can be written as follows:

$$Q_{M}^{⋆}\left(s,a\right)=E_{s^{\prime }∼P\left(\cdot |s,a\right)}\left[R\left(s,a,s^{\prime }\right)+\max_{a^{\prime }\in A}Q_{M}^{⋆}\left(s^{\prime },a^{\prime }\right)\right].$$

We can manipulate the expression to recover a new action-value function that we define as $\hat{Q}\left(s,a\right)$ :

$$\hat{Q}\left(s,a\right)=Q_{M}^{⋆}\left(s,a\right)-\Psi \left(s\right)$$

$$=E_{s^{\prime }∼P\left(\cdot |s,a\right)}\left[R\left(s,a,s^{\prime }\right)-\Psi \left(s\right)+\max_{a^{\prime }\in A}Q_{M}^{⋆}\left(s^{\prime },a^{\prime }\right)\right]$$

$$=E_{s^{\prime }∼P\left(\cdot |s,a\right)}\left[R\left(s,a,s^{\prime }\right)+\Psi \left(s^{\prime }\right)-\Psi \left(s^{\prime }\right)-\Psi \left(s\right)+\max_{a^{\prime }\in A}Q_{M}^{⋆}\left(s^{\prime },a^{\prime }\right)\right]$$

$$=E_{s^{\prime }∼P\left(\cdot |s,a\right)}\left[R\left(s,a,s^{\prime }\right)+\Psi \left(s^{\prime }\right)-\Psi \left(s\right)+\max_{a^{\prime }\in A}\left(Q_{M}^{⋆}\left(s^{\prime },a^{\prime }\right)-\Psi \left(s^{\prime }\right)\right)\right]$$ (5)

$$=E_{s^{\prime }∼P\left(\cdot |s,a\right)}\left[R^{\prime }\left(s,a,s^{\prime }\right)+\max_{a^{\prime }\in A}\left(Q_{M}^{⋆}\left(s^{\prime },a^{\prime }\right)-\Psi \left(s^{\prime }\right)\right)\right]$$

$$=E_{s^{\prime }∼P\left(\cdot |s,a\right)}\left[R^{\prime }\left(s,a,s^{\prime }\right)+\max_{a^{\prime }\in A}\left(\hat{Q}\left(s^{\prime },a^{\prime }\right)\right)\right].$$

The final result turns out to be the Bellman equation of $M^{\prime }$ , and by the uniqueness of the optimal action-value function $Q_{M^{\prime }}^{⋆}$ , we can now prove that the optimal policy of $M^{\prime }$ is still optimal for M .

$$\pi_{M^{\prime }}^{⋆}\left(s\right)\in \underset{a\in A}{\mathrm{argmax}}Q_{M^{\prime }}^{⋆}\left(s,a\right)=\underset{a\in A}{\mathrm{argmax}}Q_{M}^{⋆}\left(s,a\right)-\Psi \left(s\right).$$

Since Ψ(s) only depends on the state s , it does not affect the action selection, thus completing the proof.

### 3.5 Rank-preserving policy-assessment metric

Comparing the performance and behaviors emergent by training with different rewards needs an external, unbiased assessment metric because each reward formulation has its own scale. To this end, we introduce a *rank-preserving policy-assessment metric* (PAM) F , capturing the logical satisfaction of various requirements and evaluating the episode according to the task satisfaction.

The rank defined in Definition 1 does not capture any preferences between episodes satisfying the same class of requirements. According to our task semantics, we prefer the episode with more frequent satisfaction of the comfort requirements. We formalize it in the definition of the PAM F that we use to monitor the learning process and compare HPRS to state-of-the-art approaches.

Let $\Phi =\Phi_{S}⊎\Phi_{T}⊎\Phi_{C}$ be the set of requirements defining the task. Then, we define F as follows:

$$F\left(\Phi ,\tau \right)=\sigma \left(\Phi_{S},\tau \right)+\frac{1}{2}\sigma \left(\Phi_{T},\tau \right)+\frac{1}{4}\sigma_{\mathrm{avg}}\left(\Phi_{C},\tau \right),$$ (6)

where σ(Φ,τ) ∈ {0,1} is the satisfaction function evaluated over Φ and τ . We also define a time-averaged version for any comfort requirement φ=encouragef(s)≥0 as follows:

$$\sigma_{\mathrm{avg}}\left(\varphi ,\tau \right)=\overset{\left|\tau \right|}{\underset{i=1}{\sum }}\frac{1_{\geq 0}\left(f\left(s_{i}\right)\right)}{\left|\tau \right|}.$$ (7)

Its set-wise extension computes the set-based average.

Theorem 1 Given a task (Φ,≺) , the defined metric F preserves the episode rank such that

$$rank\left(\Phi ,\tau_{1}\right)<rank\left(\Phi ,\tau_{2}\right)\;\Rightarrow \;F\left(\Phi ,\tau_{1}\right)>F\left(\Phi ,\tau_{2}\right).$$

Proof: To prove that the metric F is a rank-preserving function (Veer et al., 2023), let us consider any episodes $\tau_{1},\tau_{2}$ , for which $rank\left(\Phi ,\tau_{1}\right)<rank\left(\Phi ,\tau_{2}\right)$ with respect to the task specification (Φ,≺) .

Let k be the first class of requirement, for which $\sigma \left(\Phi_{C_{k}},\tau_{1}\right)>\sigma \left(\Phi_{C_{k}},\tau_{2}\right)$ . We can decompose the episode evaluations in Equation 6 as follows:

$$F\left(\Phi ,\tau_{1}\right)=r_{0}+{\frac{1}{2}}^{k-1}+r_{1},$$

$$F\left(\Phi ,\tau_{2}\right)=r_{0}+0+r_{2},$$

where $r_{0},r_{1},r_{2}$ are non-negative constants. The decomposition considers that all the classes before k are evaluated in the same way for $\tau_{1}$ and $\tau_{2}$ , summing up to a constant term $r_{0}$ ; the k -th class is evaluated 1 for $\tau_{1}$ and 0 for $\tau_{2}$ , and all successive classes of requirements account $r_{1}$ and $r_{2}$ for $\tau_{1}$ and $\tau_{2}$ , respectively.

Removing the common term $r_{0}$ , it remains to prove that

$$r_{2}<{\frac{1}{2}}^{k-1}+r_{1}.$$

We use the fact that for any τ , σ(Φ,⋅)∈[0,1] and $\sigma_{\mathrm{avg}}\left(\Phi ,\cdot \right)\in \left[0,1\right]$ to upperbound $r_{2}$ as follows:

$$r_{2}=\overset{N-1}{\underset{i=k+1}{\sum }}{\frac{1}{2}}^{i-1}\sigma \left(\Phi_{C_{i}},\tau_{2}\right)+{\frac{1}{2}}^{N-1}\sigma_{\mathrm{avg}}\left(\Phi_{C},\tau_{2}\right)<\overset{N}{\underset{i=k+1}{\sum }}{\frac{1}{2}}^{i-1}.$$

Finally, we expand the geometric series as follows:

$$\overset{N}{\underset{i=k+1}{\sum }}{\frac{1}{2}}^{i-1}=\frac{{\frac{1}{2}}^{k}-{\frac{1}{2}}^{N}}{1-\frac{1}{2}}={\frac{1}{2}}^{k-1}\left(1-{\frac{1}{2}}^{N}\right)<{\frac{1}{2}}^{k-1},$$

where the last step follows by the fact that $\left(1-{\frac{1}{2}}^{N}\right)<1$ , fulfilling the proof.

**FIGURE 4:**
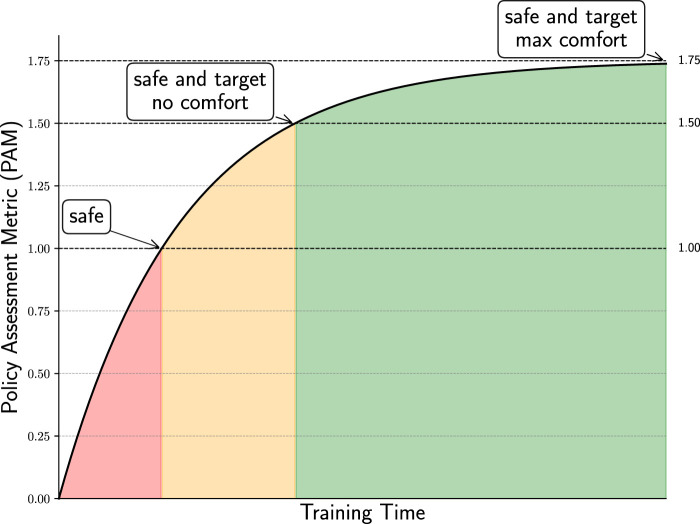
Policy assessment metric: example of a learning curve evaluated using the policy assessment metric (PAM). The curve is divided into three distinct intervals, each corresponding to different levels of task satisfaction: safety only, safety with target but poor comfort, and safety with target and maximum comfort. We use PAM as the evaluation metric because it effectively captures and distinguishes the quality of agent behavior across multiple objectives.

Since this metric is going to be adopted in the subsequent experimental section, we depict the levels of requirements’ satisfaction in Figure 4 to highlight the rank-preserving nature of PAM. Moreover, the following corollary formalizes the quantitative relations that follow from the construction of the PAM F and the semantics of the task satisfaction defined in Equation 1.

Corollary 2 Consider a task (Φ,≺) and an episode τ *.* Then, the following relations hold for F *:*

$$F\left(\Phi ,\tau \right)\geq 1.0↔\sigma \left(\Phi_{S},\tau \right),$$

$$F\left(\Phi ,\tau \right)\geq 1.5↔\sigma \left(\Phi ,\tau \right).$$

## 4 Auto-shaping library

**FIGURE 5:**
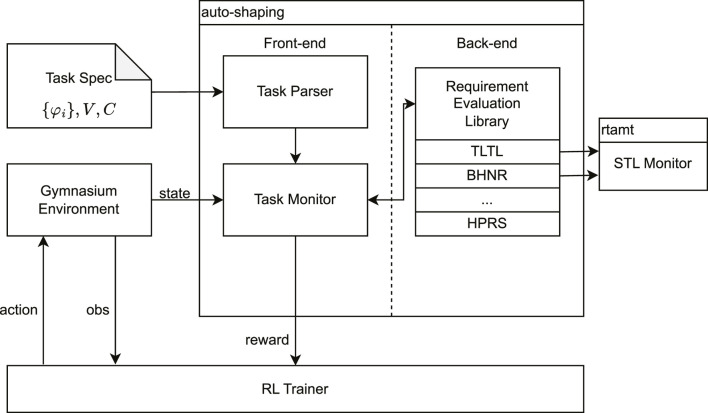
Auto-shaping library: architecture of auto-shaping, a software library designed for automatic reward shaping from hierarchical task specifications. The library accepts the environment and a formal task specification, which define the operational context and the desired behavior. It consists of a *parser* to interpret task specifications, a *monitor* to track agent performance, and various *shaping techniques*, including the proposed HPRS. The library adopts standard RL interfaces for easy integration with existing training libraries, facilitating automatic reward shaping for formal task specifications.

We implemented the proposed HPRS in the auto-shaping library. The library is implemented in Python for automatic reward generation based on declarative task specifications. It wraps the given environment to calculate rewards by evaluating the task requirements, according to the defined hierarchy. The library, depicted in Figure 5, comprises three components: (1) the task specification, (2) a frontend to parse the specification and monitor the environment, and (3) a backend that computes the reward.

### 4.1 Task specification

The task is specified using the declarative language described in Section 3.2. The users define a list of requirements, variables (V) and constants (C), and the partially ordered sets are inferred from their classes. Specifications for standard environments are provided as YAML files.

### 4.2 Parsing and online preprocessing

We use Lark to translate the task specification from textual form to a parse tree, according to the specified requirement grammar. During each step of the environment, the task monitor computes the variables defined in the task specification from the simulation state. These observable quantities are then passed to the backend for reward signal computation.

### 4.3 Shaping library

The backend component evaluates requirement specifications based on defined semantics and is responsible for computing the reward signal. The library incorporates various automatic shaping methods derived from formal task specifications, including those described in the experimental phase.

For methods based on STL monitoring, the library uses the RTAMT monitoring tool (Ničković and Yamaguchi, 2020), which implements infinity-norm and filtering quantitative semantics. The library design focused on usability and compatibility with existing RL frameworks. It is implemented as a wrapper compatible with the gymnasium API (Towers et al., 2023) and follows the standardized interaction between the agent and the environment. This design choice ensures compatibility with various RL frameworks and access to state-of-the-art algorithm implementations without requiring custom implementations. Examples are provided using stable-baselines3 (Raffin et al., 2019) and CleanRL (Huang et al., 2022). We believe that this library simplifies the process of generating reward signals in RL by automating the evaluation of hierarchical requirements from formal task specification. Its compatibility with popular RL frameworks enhances its usability and applicability, standing out as the first library providing a unified framework for reward shaping from hierarchical task specifications.

## 5 Experimental results

### 5.1 Experimental setup

To evaluate HPRS, we employ state-of-the-art implementations of RL algorithms on eight use cases: the cart-pole with an obstacle; the lunar lander with an obstacle; the bipedal walker both in the classic and hardcore versions; two customs driving tasks with single and multiple cars, respectively; and two locomotion tasks with ant and humanoid legged-robots. In each use case, we formalize a set of requirements $\Phi =\Phi_{S}⊎\Phi_{T}⊎\Phi_{P}$ and derive their partially ordered set (Φ,≺) .

To demonstrate that our reward-shaping methodology is general and completely agnostic by the underlying training algorithm, we conduct experiments using SAC from Raffin et al. (2019) and PPO from Mittal et al. (2023), which are two stable and widely adopted implementations.

For SAC, we tune the algorithm hyperparameters under the original environment and reward, starting from the configuration in rl-zoo3 (Raffin, 2020). For PPO, we use the hyperparameters as reported in Mittal et al. (2023) because they were already tuned for the tasks used in the experiments. We adopt the same configuration for each environment, and during training, we evaluate the performance of the policy at fixed intervals with respect to the aggregated PAM F and scores for each individual class. The details regarding the training and specific algorithm’s hyperparameters are reported in Table 2.

**TABLE 2:** Training configuration and hyperparameters for the simulated environments.

	CP	LL	BW	BW (hardcore)
Training and evaluation configuration				
Episode timeout	400	600	500	500
Total steps	1e6	1.5e6	2e6	3e6
Evaluation frequency	1e4	1e4	1e4	1e4
Number of evaluation episodes	10	10	10	10
Hyperparameters				
Algorithm	SAC	SAC	SAC	SAC
Discount γ	0.99	0.99	0.99	0.99
Learning rate	0.0003	0.0003	0.0003	0.0003
Buffer size	5e4	3e5	1e6	1e6
Learning starts	1e2	1e4	1e2	1e2
Batch size	64	256	256	256
Soft-update coefficient τ	0.005	0.01	0.005	0.005
Critic architecture	[256,256]	[400,300]	[256,256]	[256,256]
Policy-network architecture	[256,256]	[400,300]	[256,256]	[256,256]

	**SD**	**FLV**	**ANT**	**HUM**
Training and evaluation configuration				
Episode timeout	300	300	960	960
Total steps	1e6	1e6	1.3e8	2.6e8
Evaluation frequency	1e4	1e4	8e6	8e6
Number of evaluation episodes	10	10	30	30
Hyperparameters				
Algorithm	SAC	SAC	PPO	PPO
Discount γ	0.99	0.99	0.99	0.99
Learning rate	0.0003	0.0003	0.0005	0.0005
Buffer size	3e5	3e5	1.3e5	1.3e5
Learning starts	1e2	1e2	-	-
Batch size	256	256	32e3	32e3
Soft-update coefficient τ	0.005	0.005	-	-
Critic architecture	[256,256]	[256,256]	[400,200,100]	[400,200,100]
Policy-network architecture	[256,256]	[64,64]	[400,200,100]	[400,200,100]

### 5.2 Use cases

In the remainder of the presentation, we organize the experiments based on the type of environments into *classic-control* environments and *physics-simulated* environments. Here, we describe the benchmark tasks we used to conduct the experimental evaluation following this organization.

#### 5.2.1 Classic-control environments

We present the tasks defined over standard classic-control benchmarks. Table 3 reports all the requirements formalized in the proposed specification language for each of the use cases.

**TABLE 3:** Formalized requirements for all the tasks.

Task	Req id	Formula id	Formula
CP	Req1	$\varphi_{1}$	conquerd(s,G)=0
	Req2	$\varphi_{2}$	$ensure\;|x|\leq x_{\lim}$
	Req3	$\varphi_{3}$	$ensure\;|\theta |\leq \theta_{\mathrm{fall}}$
	Req4	$\varphi_{4}$	ensured(s,O)>0
	Req5	$\varphi_{5}$	$encourage\;|\theta |\leq \theta_{\mathrm{balance}}$
LL	Req1	$\varphi_{1}$	conquerd(s,G)=0
	Req2	$\varphi_{2}$	ensured(s,O)≥0
	Req3	$\varphi_{3}$	$ensure\;|x|\leq x_{\text{lim}}$
	Req4	$\varphi_{4}$	$encourage\;|\theta |\leq \theta_{\text{comf}}$
	Req5	$\varphi_{5}$	$encourage\;|\dot{\theta }|\leq {\dot{\theta }}_{\text{comf}}$
BW	Req1	$\varphi_{1}$	achieved(s,G)=0
	Req2	$\varphi_{2}$	ensured(s,O)>0
	Req3	$\varphi_{3}$	$encourage\;|\theta |\leq \theta_{\text{comf}}$
	Req4	$\varphi_{4}$	$encourage\;|\dot{\theta }|\leq {\dot{\theta }}_{\text{comf}}$
	Req5	$\varphi_{5}$	$encourage\;|\dot{x}|\geq v_{\text{min}}$
	Req6	$\varphi_{6}$	$encourage\;|\dot{y}|\leq {\dot{y}}_{\text{comf}}$
SD	Req1	$\varphi_{1}$	$ensure\;d_{\text{walls}}\left(s\right)>0$
	Req2	$\varphi_{2}$	achieveL(s)=1.0
	Req3	$\varphi_{3}$	$encourage\;d_{\text{center}}\left(s\right)\leq d_{\text{comf}}$
	Req4	$\varphi_{4}$	$encourage\;v\geq v_{\text{min}}$
	Req5	$\varphi_{5}$	$encourage\;v\leq v_{\text{max}}$
	Req6	$\varphi_{6}$	$encourage\;|\alpha |\leq \alpha_{\text{comf}}$
	Req7	$\varphi_{7}$	encourage|a|≤Δa
FLV	Req1	$\varphi_{1}$	achieveL(s)=1.0
	Req2	$\varphi_{2}$	$ensure\;d_{\text{walls}}\left(s\right)>0$
	Req3	$\varphi_{3}$	$ensure\;d_{\text{lead}}\left(s\right)>0$
	Req4	$\varphi_{4}$	$encourage\;d_{\text{lead}}\left(s\right)\geq d_{\text{min, comf}}^{\text{lead}}$
	Req5	$\varphi_{5}$	$encourage\;d_{\text{lead}}\left(s\right)\leq d_{\text{max, comf}}^{\text{lead}}$
	Req6	$\varphi_{6}$	$encourage\;|\alpha |\leq \alpha_{\text{comf}}$
	Req7	$\varphi_{7}$	encourage|a|≤Δa
ANT	Req1	$\varphi_{1}$	achieved(s,G)=0
	Req2	$\varphi_{2}$	$ensure\;h_{\text{body}}>h_{\text{min}}$
	Req3	$\varphi_{3}$	$encourage\;v\geq v_{\text{min}}$
	Req4	$\varphi_{4}$	$encourage\;|\theta_{\text{goal}}|\leq \theta_{\text{comf}}$
	Req5	$\varphi_{5}$	$encourage\;\|a{\|}_{2}\leq a_{\text{comf}}$
	Req6	$\varphi_{6}$	$encourage\;p_{\text{joint}}\leq p_{\text{lim}}$
HUM	Req1	$\varphi_{1}$	achieved(s,G)=0
	Req2	$\varphi_{2}$	$ensure\;h_{\text{body}}>h_{\text{min}}$
	Req3	$\varphi_{3}$	$encourage\;v\geq v_{\text{min}}$
	Req4	$\varphi_{4}$	$encourage\;|\theta_{\text{goal}}|\leq \theta_{\text{comf}}$
	Req5	$\varphi_{5}$	$encourage\;\|a{\|}_{2}\leq a_{\text{comf}}$
	Req6	$\varphi_{6}$	$encourage\;p_{\text{joint}}\leq p_{\text{lim}}$
	Req7	$\varphi_{7}$	$encourage\;g_{\text{vert}}\geq t_{\text{up}}$

##### 5.2.1.1 Cart-pole (CP) with obstacle

A pole is attached to a cart that moves between a left and a right limit within a flat and frictionless environment. Additionally, the environment has a target area within the limits and a static obstacle standing above the track. The system is controlled by applying a continuous force to the cart, allowing the left and right movements of the cart-pole with different velocities. In order to reach the goal and satisfy the target requirement, the cart-pole must perform an uncomfortable and potentially unsafe maneuver: since moving a perfectly balanced pole would result in a collision with the obstacle, the cart-pole must lose balancing and pass below it.

We formulate *three safety* requirements and *one target* and *one comfort* requirement, which are defined using the following constants: (1) G —the coordinates of the goal, (2) O —the area that is occupied by the obstacle, (3) $x_{lim}$ —the limit of the world, (4) $\theta_{fall}$ —the limit of the angle of the pole, and (5) $\theta_{balance}$ —the maximum angle that we consider as balancing.

##### 5.2.1.2 Lunar lander (LL) with obstacle

It consists of a variation in the original lunar lander environment, where the agent controls a lander with the objective to land at the pad with coordinates (0,0). We add an obstacle to the environment in the vicinity of the landing pad, which makes the landing task harder because, during the navigation, the agent has to avoid it to reach the pad. Landing outside of the pad is also possible but at the cost of not achieving the task. We allow continuous actions to control the lander engine.

We formulate *two safety*, *one target*, and *two comfort* requirements, which are defined using the following constants: (1) G —the coordinates of the landing area, (2) O —the area that is occupied by the static obstacle, (3) $x_{lim}$ —the limit of the world, (4) $\theta_{comf}$ —the maximum comfortable angle, and (5) ${\dot{\theta }}_{comf}$ —the maximum comfortable angular velocity.

##### 5.2.1.3 Bipedal walker (BW)

The robot’s objective is to move forward toward the end of the field without falling. We consider two variants of this case study: the classical one with the flat terrain and the hardcore one with holes and obstacles.

We consider the same task specification for both the versions, consisting of *one safety*, *one target*, and *four comfort* requirements to encourage keeping the hull balance and avoiding oscillations. To formalize the task described, we define the following constants: (1) O —the set of coordinates occupied by the static obstacle, (2) $\theta_{comf}$ —the maximum comfortable angle, (3) ${\dot{\theta }}_{comf}$ —the maximum comfortable angular velocity, and (4) ${\dot{y}}_{comf}$ —the maximum comfortable vertical velocity.

#### 5.2.2 Physics-simulated environments

We present the tasks defined over environments using advanced physics-based simulators. In particular, we simulated driving tasks in PyBullet (Erwin and Yunfei, 2016) and robotics locomotion tasks in Isaac-Sim (NVIDIA, 2023). Table 3 reports all the requirements formalized in the proposed specification language for each of the use cases.

##### 5.2.2.1 Safe driving (SD)

The task consists of a car driving in a closed-loop track by end-to-end control of the speed and steering angle. The details of this use case are presented as a motivating example in Section 1.2.

##### 5.2.2.2 Follow leading vehicle (FLV)

It consists of the extension with a non-controllable leading vehicle, which the car aims to safely follow, keeping a comfortable distance to it. The agent does not access the full state but only the most-recent observations from LiDAR, noisy velocity estimates, and previous controls. The safety requirements are extended to consider the collision with the leading vehicle, and the comfort requirements consider the control requirements and encourage the car to keep a comfortable distance without any constraints on the car speed.

We formulate *two safety*, *one target*, and *four comfort* requirements. To specify them, we build on the quantities introduced in the safe driving task and the one described in the example. We additionally defined the following comfort requirements: encouraging a small steering angle (α) , smooth controls (|a|) , and the agent to keep a distance between $\left[d_{\text{min},comf}^{lead},d_{\text{max},comf}^{lead}\right]$ .

##### 5.2.2.3 Ant (ANT)

The robot is a four-legged robot that moves in a plane. The goal consists of reaching a goal position, and the ant has control of its eight joints to efficiently walk forward while maintaining stability.

We formulate *one safety*, *one target*, and *four comfort* requirements, using the following constants: (1) G —the coordinates of the goal, (2) $h_{\text{min}}$ —the minimum height of the torso under which the robot is considered to fall down, (3) $v_{\text{min}}$ —the desired minimum speed in the direction of the goal, (4) $\theta_{\text{comf}}$ —the tolerable deviation of the robot heading to the goal, (5) $a_{\text{comf}}$ —the upperbound on the action norm to encourage energy-efficient gaits, and (6) $p_{\text{lim}}$ —the limit in joint position.

##### 5.2.2.4 Humanoid (HUM)

The task is the same as the ant, but the robot is a more complex bipedal robot that resembles a human figure. The goal is to control the torque applied to 17 joints to walk toward a target while maintaining balance.

We formulate *one safety*, *one target*, and *five comfort* requirements. In addition to the quantities introduced in the ant, we defined a comfort requirement on $g_{\text{vert}}$ , which is the projection of the base up vector onto the vertical axis. To encourage an upright posture, we reward it to be above $t_{\text{up}}$ .

### 5.3 Reward baselines

We implemented HPRS as in Equation 4. To answer **RQ1** and **RQ2**, we compared it with the original reward defined by experts in each environment, which is indicated as *Shaped*, and three additional baselines from state-of-the-art work:

• *TLTL* (Li et al., 2017) specifies tasks in a bounded (truncated) LTL variant equipped with an infinity-norm quantitative semantics (Maler and Nickovic, 2004). The task specification is a conjunction of the task requirements, and its quantitative evaluation of the episode is used as an episodic return. We employ the state-of-the-art RTAMT monitoring tool to compute the episode robustness (Ničković and Yamaguchi, 2020). We choose this baseline because it represents the most natural way to adopt formal task specifications to shape a reward signal, directly adopting logic quantitative semantics as a return.
• *BHNR* (Balakrishnan and Deshmukh, 2019) specifies tasks in a fragment of signal temporal logic (STL) consisting of safety and liveness formulas. However, since the infinity-norm quantitative semantics adopted in STL and TLTL faces *locality* and *masking* (Mehdipour et al., 2020) due to episodic evaluation and min/max operators, respectively, the authors propose an alternative formulation. BHNR adopts a filtering semantics (Rodionova et al., 2016) and uses a sliding-window approach to produce more frequent feedback to the agent. At each step, it uses the quantitative semantics to evaluate a sequence of H states. We choose this baseline because it still adopts a formal language to specify the task while mitigating some of the limitations of prior logic-based approaches.
• *MORL* (Brys et al., 2014) implements the multi-objectivization of the task and solves the multi-objective problem by linear scalarization. Treating each requirement as an objective, it independently evaluates them and linearly combines the individual rewards. We choose this baseline to benchmark against a standard multi-objective approach, and it allows us to analyze the impact of using the adaptive weighting scheme proposed in HPRS. To further assess the sensitivity to the choice of weights, we consider two variants: uniform weights *MORL (unif.)*, where we use a unit weight for each requirement (i.e., w=1 ), and decreasing weights *MORL (decr.)*, where safety is more important than the target and the target is more important than comfort. In the latter, we decrease the weight by halving them for each class (i.e., w=1.0 for safety, w=0.5 for target, and w=0.25 for comfort).

### 5.4 Experimental evaluation

#### 5.4.1 Comparison with baselines

We compare *HPRS* with the above baselines to answer **RQ1**. We empirically assess the training performance in terms of training efficiency and alignment with the desired requirements.

For a sound and unbiased comparison and for accounting to the different reward ranges of the baselines, we use the PAM F defined in Section 3.5. F allows categorizing each episode τ as (1) satisfying safety if F(Φ,τ) ≥ 1 , (2) satisfying safety and target if F(Φ,τ) ≥ 1.5 , and (3) additionally maximizing comfort if F(Φ,τ) is close to 1.75. We emphasize that F is not used for training. Hence, it should not be used to evaluate the convergence of the RL algorithm in the training process.

**FIGURE 6:**
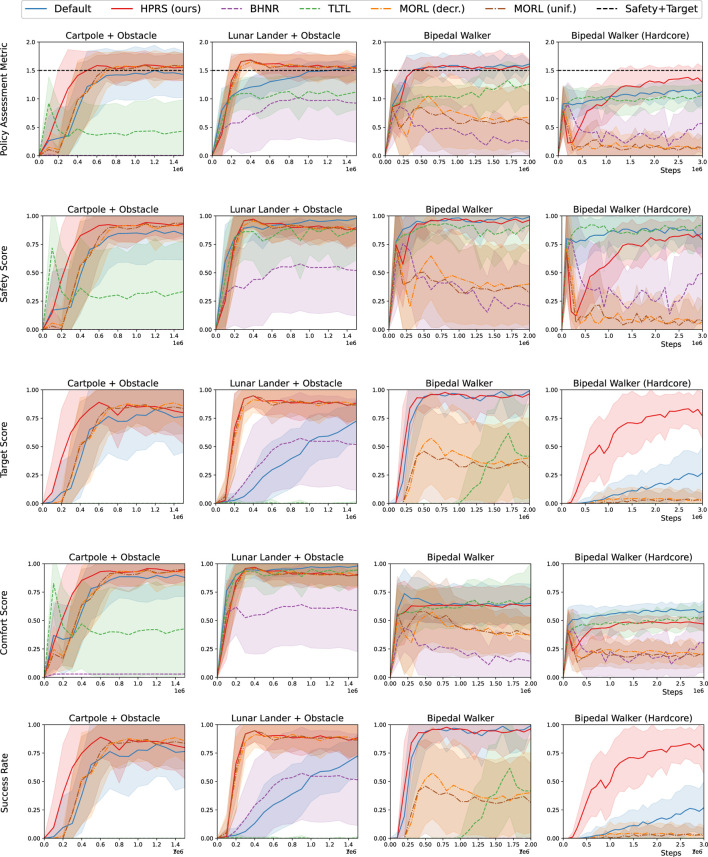
Training in classic-control environments: performance for various reward-shaping techniques, including logic-based (*TLTL*, *BHNR*, and *HPRS*), multi-objective (*MORL(unif.)* and *MORL(dec.)*), and engineered design (*shaped*). We report **(row 1)** the rank-preserving PAM, (**rows 2–4**) the scores for individual classes of requirements, and (**row 5**) the success rate of the overall task. Performance are reported as mean (*solid curve*) and the standard deviation (*shadow*) over 10 seeds.

**FIGURE 7:**
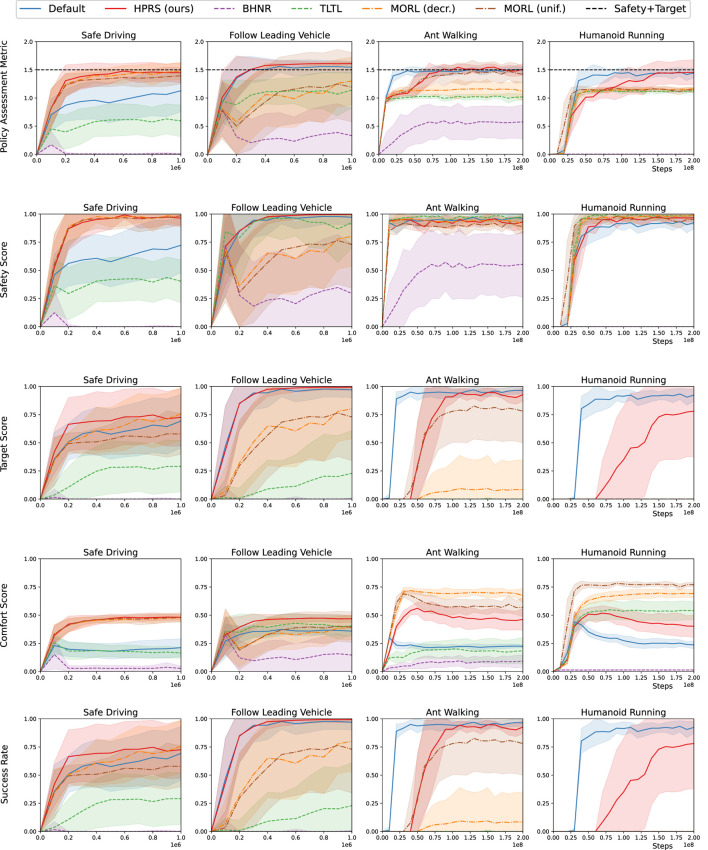
Training in physics-simulated environments: performance for various reward-shaping techniques, including logic-based (*TLTL*, *BHNR*, and *HPRS*), multi-objective (*MORL(unif.)* and *MORL(dec.)*), and engineered design (*shaped*). We report **(row 1)** the rank-preserving PAM, (**rows 2–4**) the scores for individual classes of requirements, and (**row 5**) the success rate of the overall task. Performance are reported as mean (*solid curve*) and the standard deviation (*shadow*) over 10 seeds.

Figures 6, 7 show that HPRS has superior performance, as indicated by faster convergence to task-satisfying policies reaching the same (and often better) level of performance of the shaped reward in all the tasks. The other approaches are not competitive to learn a policy for tasks with a high number of requirements.

#### 5.4.2 Offline evaluation of the learned behaviors

Regardless of the utility of a custom sound evaluation metric, capturing complex behaviors and evaluating the satisfaction of the hierarchical structure of requirements with a single scalar remains challenging. For this reason, to answer **RQ2**, we perform an extensive offline evaluation by comparing the policies (agents) trained with HPRS against those trained by using the other baseline rewards. Furthermore, we provide evidence of the emergent behaviors in the submitted video.

We evaluate each trained policy in 50 random episodes for a total of 500 episodes for each task. Table 4 reports the success rate for incremental sets of safety (S), safety and target (S + T), and safety, target, and comfort (S + T + C).

**TABLE 4:** Offline evaluation of trained agents: we evaluate the agents trained with different reward-shaping techniques, collecting 50 different simulations for each agent, starting from the same initial conditions. We report (**S**) the rate of episodes with all safety requirements satisfied, (**S + T**) the rate of episodes where both safety and target requirements are met, and (**S + T + C**) the rate for safety and target weighted by the satisfaction of comfort requirements. Results within <5% of the best-performing reward shaping are marked, indicating comparable performance.

Environment	Reward	S	S + T	S + T + C
		Succ.Rate (%)	Succ.Rate (%)	Succ.Rate (%)
Cart-pole	Shaped	0.87	0.78	0.75
	TLTL	0.31	0.00	0.00
	BHNR	0.00	0.00	0.00
	MORL (unif.)	0.94	0.90	**0.87**
	MORL (decr.)	0.88	0.78	0.75
	**HPRS(ours)**	0.92	0.87	**0.84**
Lunar lander	Shaped	0.98	0.72	0.72
	TLTL	0.92	0.00	0.00
	BHNR	0.51	0.49	0.49
	MORL (unif.)	0.91	0.91	**0.90**
	MORL (decr.)	0.94	0.91	**0.91**
	**HPRS(ours)**	0.91	0.91	**0.89**
Bipedal walker	Shaped	0.99	0.99	**0.51**
	TLTL	0.96	0.45	0.27
	BHNR	0.21	0.00	0.00
	MORL (unif.)	0.40	0.40	0.19
	MORL (decr.)	0.43	0.43	0.20
	**HPRS(ours)**	0.96	0.96	**0.48**
Bipedal walker (hardcore)	Shaped	0.84	0.29	0.17
	TLTL	0.98	0.00	0.00
	BHNR	0.55	0.00	0.00
	MORL (unif.)	0.07	0.03	0.02
	MORL (decr.)	0.06	0.03	0.02
	**HPRS(ours)**	0.85	0.85	**0.44**
Safe driving	Shaped	0.74	0.74	0.20
	TLTL	0.38	0.32	0.10
	BHNR	0.00	0.00	0.00
	MORL (unif.)	0.99	0.69	**0.32**
	MORL (decr.)	0.96	0.75	**0.35**
	**HPRS(ours)**	0.97	0.73	**0.33**
Follow leading vehicle	Shaped	0.97	0.97	0.34
	TLTL	0.94	0.12	0.06
	BHNR	0.31	0.00	0.00
	MORL (unif.)	0.74	0.73	0.35
	MORL (decr.)	0.82	0.81	0.37
	**HPRS(ours)**	1.00	0.99	**0.46**
Ant	Shaped	0.96	0.96	0.23
	TLTL	0.97	0.0	0.00
	BHNR	0.43	0.0	0.00
	MORL (unif.)	0.85	0.73	**0.44**
	MORL (decr.)	0.94	0.07	0.05
	**HPRS (ours)**	0.90	0.90	**0.44**
Humanoid	Shaped	0.99	0.99	0.23
	TLTL	0.99	0.00	0.00
	BHNR	0.0	0.00	0.00
	MORL (unif.)	0.88	0.00	0.00
	MORL (decr.)	0.95	0.00	0.00
	**HPRS (ours)**	0.99	0.74	**0.28**

The results show that policies learned with HPRS consistently complete the task in most evaluations, proving their ability in trading-off the different requirements and reaching the same level of performance of handcrafted rewards despite being automatically derived from declarative specifications. Although other baselines struggle in capturing the correct objective and do not show consistent performance across different domains, we highlight that HPRS is <5% close to the best-performing approach in all the tasks.

Logic-based approaches, such as *TLTL* and *BHNR*, consider the task as a unique specification and result in policies that either eagerly maximize the progress toward the target, resulting in unsafe behaviors, or converge to an over-conservative behavior that never achieves task completion. This observation highlights the weakness of these approaches when dealing with many requirements because the dominant requirement could mask out the others, even if normalized adequately to the signal domain.

Multi-objective approaches are confirmed to be sensitive to weight selection. Their performance is competitive in some of the tasks, but they perform poorly in more complex tasks, such as the bipedal walker.

Finally, the shaped reward results in policies capturing the desired behavior, confirming the good reward shaping proposed in the original environments. However, considering the current training budget, HPRS produces a more effective learning signal, resulting in better-performing policies. Although logic-based shaping approaches, such as TLTL and BHNR, consistently underperformed compared to shaped rewards and multi-objective shaping (MORL) demonstrates decreasing performance in tasks with long horizons, our proposed HPRS method consistently demonstrates stable performance across all specified tasks.

#### 5.4.3 Ablation study on the hierarchical task structure

We evaluate the impact of individual requirements on the hierarchical structure of HPRS to understand their contribution on the emergent behavior and answer **RQ3**.

We focus on the comfort requirements that have the least priority and, thus, the minor influence on the value of the final reward. Specifically, we study how the comfort requirements improve the observed comfort. We set up an ablation experiment on them and compare the performance of the resulting policies.

Table 5 reports the evaluation for policies trained with (+comfort) and without (-comfort) comfort requirements.

**TABLE 5:** Ablation on comfort requirements: we evaluate the agents trained with HPRS using the full hierarchy of requirements (**+Comfort**) and the hierarchy excluding comfort (**-Comfort**). The satisfaction of comfort requirements uses the time-averaged satisfaction (Equation 5). The results report mean and standard deviation over 50 evaluation episodes for each agent.

	+Comfort	-Comfort
Cart-pole	σ_avg_	σ_avg_
Keep the balance	1.00±0.00	1.00±0.00
Lunar lander		
Hull angle	0.99±0.05	0.99±0.02
Hull angular velocity	0.97±0.07	0.98±0.05
Bipedal walker		
Hull angle	0.80±0.17	0.33±0.27
Hull angular velocity	1.00±0.00	0.99±0.01
Vertical oscillation	0.98±0.01	0.91±0.11
Horizontal velocity	0.95±0.01	0.92±0.03
Bipedal walker hardcore		
Hull angle	0.70±0.13	0.29±0.14
Hull angular velocity	1.00±0.00	0.99±0.01
Vertical oscillation	0.83±0.06	0.75±0.09
Horizontal velocity	0.94±0.05	0.81±0.10
Safe driving	σ_avg_	σ_avg_
Keep the center	0.39±0.12	0.33±0.10
Min velocity	0.48±0.21	0.89±0.06
Max velocity	0.99±0.01	0.36±0.14
Comfortable steering	0.27±0.08	0.08±0.04
Smooth control	0.70±0.07	0.32±0.07
Follow leading vehicle		
Min distance	0.55±0.22	0.85±0.16
Max distance	0.90±0.08	0.61±0.18
Comfortable steering	0.23±0.05	0.15±0.04
Smooth control	0.78±0.09	0.41±0.11
Ant		
Forward velocity	0.00±0.00	0.04±0.06
Heading to target	0.62±0.23	0.27±0.20
Action norm	0.59±0.21	0.02±0.02
Joints within limits	0.55±0.20	0.01±0.01
Humanoid		
Forward velocity	0.01±0.03	0.0±0.0
Heading to target	0.65±0.23	0.37±0.32
Upright posture	0.50±0.20	0.35±0.31
Action norm	0.76±0.22	0.42±0.22
Joints within limits	0.10±0.09	0.0±0.0

As in the offline evaluation, we collect 50 episodes for each seed and compute the ratio of satisfaction of comfort requirements over each episode, according to the time-average defined in Equation 7.

In all the tasks, introducing comfort requirements positively impacts the evaluation. Although some of the requirements are almost always satisfied by both configurations, the satisfaction of other requirements significantly improves once comfort rules are introduced, which is denoted by an increase in the mean satisfaction and a reduction in its standard deviation. In particular, in the driving tasks, the smaller steering magnitude and smoother transition between consecutive controls make the policy amenable to transfer to real-world, as demonstrated in the next section.

## 6 Real-world demonstration

In this section, we answer **RQ4** and describe the real-world experiments conducted to validate the usability of HPRS on robotics systems. Specifically, we trained driving policies with HPRS and evaluated their performance in a real-world setting using 1/10th-scaled vehicles of the F1TENTH series (O’Kelly et al., 2020). F1TENTH provides an affordable yet sophisticated platform for development, encompassing all the necessary hardware and software components of an autonomous driving car.

### 6.1 Training

**FIGURE 8:**
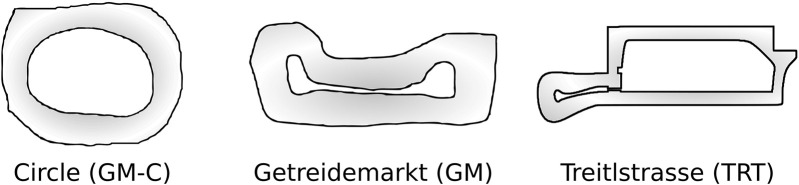
Racing tracks used for training and deployment of the driving agent.

To train the driving policies, we used the racecar_gym environment (Brunnbauer et al., 2022), which builds on the Bullet physics simulator (Erwin and Yunfei, 2016). The environment provides a realistic 3D simulation of the F1TENTH vehicles, including their sensor suite and actuation capabilities. In particular, the agent observes sensor readings from LiDAR and velocity and controls the car by setting a target velocity and steering angle. To account for the lack of full-state observability, we stacked the most recent k observations and actions (k=3) . The training tracks, shown in Figure 8, were physically created at our laboratory facilities. The tracks were then mapped with Cartographer SLAM (Hess et al., 2016) and imported into the simulator to closely mimic the real-world environment.

For the safe driving task, we trained the policy on both the GM (21.25,m) and TRT (51.65,m) tracks and deployed it in the GM track. The GM track is a narrow track with multiple tight turns, resembling the (scaled) layout of the track used in the head-to-head final of the F1TENTH Autonomous Grand-Prix 2021 in Prague. The TRT track, on the other hand, is characterized by long straightaways and 90-degree curves, and we keep it as a reference from the simulation environment (Brunnbauer et al., 2022). By training on both tracks, we enforce robustness to variations in track design and layout. For the follow lead vehicle task, we trained and deployed the policy on the GM-C (13.50,m) track, which is a modified version of the GM track that includes a lead vehicle for the ego vehicle to follow.

During training, we sampled the track and the car pose at random. To train a policy that was robust to environmental mismatch between simulation and real-world settings, we performed domain randomization (Tobin et al., 2017) of the simulation parameters. This involved randomizing the values of various simulation parameters, including the noise characteristics of the sensors and the gains of the actuators. The randomization intervals are reported in Table 6. This approach helped the policy generalize well to real-world settings, where the exact values of these parameters may differ from those in the simulator.

**TABLE 6:** Domain randomization parameters: randomization intervals for physical parameters were used during training to improve sim-to-real transfer of the trained agent. For each parameter, the interval specifies the range of values sampled from the given distribution. These randomizations create diverse training environments, enabling the agent to transfer better in real-world scenarios.

Component	Physical parameter	Randomization interval	Units	Distribution
Actuator	Steering multiplier	[0.5, 0.75]	-	Uniform
Actuator	Velocity multiplier	[20, 25]	-	Uniform
Actuator	Maximum velocity	[3.5, 4.0]	m/s	Uniform
Sensor	Velocity noise (std dev)	[0.01, 0.05]	m/s	Uniform

### 6.2 Deployment

To validate the performance of our trained policies in a real-world setting, we used 1/10th-scaled vehicles from the F1TENTH series (O’Kelly et al., 2020). The hardware platform consisted of an off-the-shelf Traxxas Ford Fiesta ST race car chassis, which was actuated by a brushless DC electric motor and controlled by a VESC 6 MkIV electronic speed controller. We used a Hokuyo UST-10LX 2D LiDAR sensor to sense distances to surrounding obstacles and walls. This sensor has a field of view of 270° and can scan up to 10 m with high precision. We also read a noisy estimate of the vehicle’s velocity directly from the VESC. All model inputs, including the LiDAR scan, VESC data, and last actions, were normalized to lie within the range ±1 .

We integrated the trained agent into a ROS node within the F1TENTH software setup, with speed and steering commands passed to the auxiliary nodes from the F1TENTH software stack. These nodes automatically computed the motor RPM and servo position. To account for differences in sensor rates and policy inference between the simulated and real-world environments, we ran a control loop at a frequency of 10 Hz on an NVIDIA Jetson Xavier NX embedded computing platform. At each iteration, we collected the most recent readings from the sensors, prepared the data, and performed inference.

**FIGURE 9:**
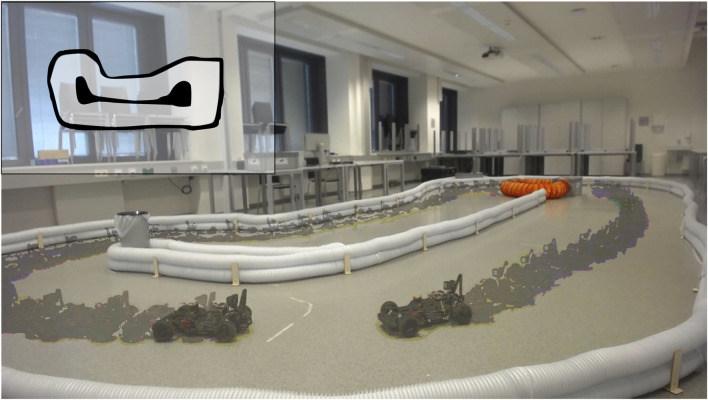
Real-world demonstration of deployment on an F1TENTH car: Successful deployment of a policy trained with HPRS on a real-world F1TENTH car, completing a full lap of the racetrack. The overlay depicts the sequence of car poses, illustrating the car’s trajectory throughout the lap. The inset image (*top-left*) shows the layout of the racetrack used in this experiment.

We tested the performance of our trained policies by deploying them in real-world scenarios. Figure 9 shows a successful deployment of our safe-driving task, where the car smoothly drives along the track. We also demonstrate the car’s ability to safely follow a leading vehicle while maintaining a comfortable distance. The video attached to this submission shows these behaviors in action.

## 7 Discussion and limitations

We have presented a principled approach for shaping rewards from a set of partially ordered formalized requirements and demonstrated its practical usability in simulated and real-world applications. Here, we consider a few limitations to the proposed approach that are worth considering for future work.

First, the continuous potential function definition relies on well-shaped signals in the requirements, as is usually the case in continuous control tasks. However, in systems with hybrid dynamics and discrete jumps, this may not hold. Despite advancements in the exploration strategy for RL, discrete and sparse signals inherently complicate exploration.

Second, addressing a high number of conflicting requirements remains an open challenge. We handle this by assigning priorities among requirements, yielding positive results for up to seven requirements across diverse applications. This demonstrates the applicability of the proposed methodology, reducing the number of design choices that must be made for each problem. However, the problem of scalarization remains a fundamental issue in multi-objective optimization research.

Furthermore, we validate our approach by deploying the trained policy on hardware in two autonomous driving tasks. However, the transfer from simulation to hardware is a well-known challenge, extending beyond our study. To address this, we close the simulation-to-real mismatch using a physics simulator with realistic noisy LiDAR and velocity sensors. We match control frequency to hardware capabilities and train a robust policy with domain randomization of physics parameters. Despite these efforts, sim-to-real transfer remains challenging and requires expertise for accurate modeling of the system parameters.

Finally, we re-implemented the baselines for compatibility with the latest RL frameworks. When striving for fidelity to original formulations and keeping the implementation close to the existing codebases, the absence of a unified framework poses challenges for full reproducibility. For this reason, we release the auto-shaping library to promote a reusable and transparent approach to foster research on reward design and alignment in the training of AI technologies.

## 8 Conclusion

This paper introduced HPRS, a novel, hierarchical, potential-based reward-shaping method and tool for tasks (Φ,≺) , consisting of a partially ordered set of safety, target, and comfort requirements. We conducted experiments on eight continuous-control benchmarks, comparing HPRS to many reward-shaping techniques, including logic-based, multi-objective, and engineered solutions. In the experiments, we show that HPRS performs well in a large variety of tasks. Moreover, conducting extensive offline evaluation and ablation on the hierarchy of requirements, we show that the multivariate reward with adaptive weights based on priorities enhances comfort without compromising the satisfaction of safety and target requirements, unlike other approaches which fail to capture the interdependence among different classes of requirements. Finally, we demonstrated the real-world applicability of HPRS through two sim-to-real experiments on driving benchmarks using F1TENTH vehicles, showcasing smooth autonomous vehicle control.

The idea of automatically shaping rewards from specifications possessing an evaluation procedure is general and agnostic to the plant and the RL algorithm adopted. We demonstrate this in the experiments by training with different RL algorithms and showing that HPRS, despite being automatic and based on declarative specifications, can achieve performance comparable to engineered solutions shaped by experts. We believe that our approach can bring many benefits when learning policies for autonomous agents from a set of well-defined rules with well-known priorities. There is, nevertheless, sufficient room to consider variants of this approach. The choice of the specification language and its semantics are flexible, and any representation of requirements equipped with an evaluation function for observed behaviors can be used for hierarchical reward shaping. In this paper, we focused on *unbounded* temporal operators defined over episodic tasks.

In subsequent work, we intend to consider more expressive operators and study the formalization of requirements beyond safety, progress, and comfort, such as the ethical, legal, and performance objectives.

## Data Availability

Publicly available datasets were analyzed in this study. These data can be found at: Experiments repo: https://github.com/edalexAguilar/reward_shaping. Experiments logs: https://zenodo.org/records/7075333. Auto-shaping Library: https://github.com/luigiberducci/auto-shaping.
